# 1,1′,3,3′,5,5′-Hexamethyl­spiro­[furo[2,3-*d*]pyrimidine-6(5*H*),5′-pyrimidine]-2,2′,4,4′,6′(1*H*,3*H*,1′*H*,3′*H*,5′*H*)-penta­one

**DOI:** 10.1107/S1600536809019618

**Published:** 2009-05-29

**Authors:** Nader Noroozi Pesyan, Saeed Rastgar, Yaser Hosseini

**Affiliations:** a Department of Chemistry, Faculty of Science, Urmia University, 57159 Urmia, Iran

## Abstract

In the title mol­ecule, C_15_H_18_N_4_O_6_, the fused 2,3-dihydro­furan ring has an envelope conformation and the spiro pyrimidine ring has a half-chair conformation. In the crystal, short inter­molecular O⋯C contacts of 2.835 (4) and 2.868 (4) Å between the carbonyl groups indicate the existence of electrostatic inter­actions, which link the mol­ecules into corrugated sheets parallel to the *ab* plane.

## Related literature

For applications of furo[2,3-*d*]pyrimidine derivatives, see Cody *et al.* (1997 ▶). For a related crystal structure, see Malathy Sony *et al.* (2002 ▶).
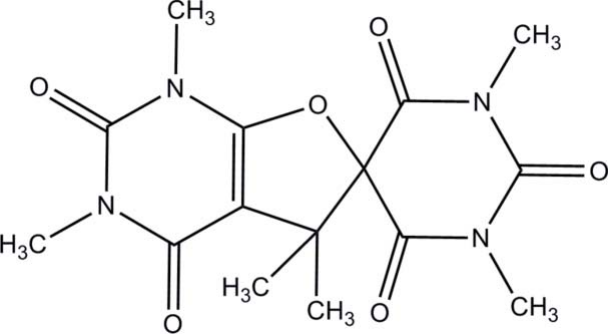


## Experimental

### 

#### Crystal data

C_15_H_18_N_4_O_6_
*M* *_r_* = 350.33
Orthorhombic, 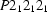

*a* = 8.0122 (9) Å
*b* = 11.9181 (14) Å
*c* = 16.4037 (19) Å
*V* = 1566.4 (3) Å^3^
*Z* = 4
Mo *K*α radiation
μ = 0.12 mm^−1^
*T* = 120 K
0.21 × 0.14 × 0.12 mm

#### Data collection

Bruker SMART 1000 CCD area-detector diffractometer
Absorption correction: multi-scan (*SADABS*; Sheldrick, 1998 ▶) *T*_min_ = 0.980, *T*_max_ = 0.989
15042 measured reflections
1964 independent reflections
1589 reflections with *I* > 2σ(*I*)
*R*_int_ = 0.041

#### Refinement

*R*[*F* ^2^ > 2σ(*F* ^2^)] = 0.047
*wR*(*F* ^2^) = 0.089
*S* = 1.01
1964 reflections
232 parameters
H-atom parameters constrained
Δρ_max_ = 0.20 e Å^−3^
Δρ_min_ = −0.22 e Å^−3^

### 

Data collection: *SMART* (Bruker, 1998 ▶); cell refinement: *SAINT-Plus* (Bruker, 1998 ▶); data reduction: *SAINT-Plus*; program(s) used to solve structure: *SHELXTL* (Sheldrick, 2008 ▶); program(s) used to refine structure: *SHELXTL*; molecular graphics: *SHELXTL*; software used to prepare material for publication: *SHELXTL*.

## Supplementary Material

Crystal structure: contains datablocks I, global. DOI: 10.1107/S1600536809019618/cv2553sup1.cif

Structure factors: contains datablocks I. DOI: 10.1107/S1600536809019618/cv2553Isup2.hkl

Additional supplementary materials:  crystallographic information; 3D view; checkCIF report

## Figures and Tables

**Table 1:** Selected interatomic distances (Å)

C8⋯O2^i^	2.835 (4)
C3⋯O5^ii^	2.868 (4)

Symmetry codes: (i) 
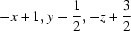
; (ii) 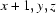.

**Fig. 1.**
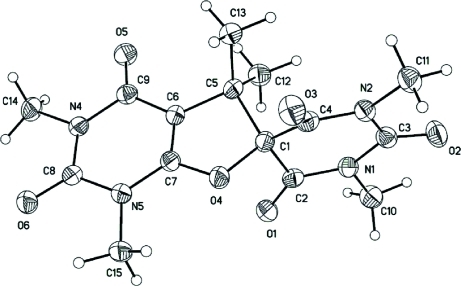
The molecular structure of the title compound showing the atomic numbering and 50% probability displacement ellipsoids.

## supplementary crystallographic information

### Comment

Fused pyrimidine compounds are valued in view of their well-known biological properties. As example, the furo[2,3-*d*]pyrimidine antifolate derivative introduced as novel classical antitumor agent (Cody *et al.*, 1997). Herewith we present the title compound, (I).

In (I) (Fig. 1),the fused 2,3-dihydrofuran ring has an envelope conformation, and spiro pyrimidine ring has a half-chair conformation. Spiro pyrimidine ring  is nearly perpendicular to 2,3-dihydro furan ring moiety, as was observed earlier in the related compound (Malathy Sony *et al.*, 2002). Torsion angles C2–C1–O4–C7 and C2–C1–C5–C6 are -99.39 (3)° and 94.87 (3)°, respectively. In the crystal, short intermolecular O···C contacts (Table 1) between the carbonyl groups prove an existing of electrostatic interactions, which link the molecules into corrugated sheets parallel to *ab* plane.

### Experimental

In a 50 ml round bottom flask (in an ice-bath) equipped with magnetic stirrer was added 200 mg (1.89 mmol) cyanogen bromide in 10 ml acetone. Then a solution of 295 mg (1.89 mmol) 1,3-dimethylbarbituric acid and 202 mg (2.00 mmol) triethylamine in acetone was added drop wise by reparatory funnel during 1 h. The white solid precipitated after few minutes and the color of liquid turned red. Initially, the precipitate was dissolved in acetone. A white crystalline colorless solid was formed after allowing the solution to stand overnight (228 mg, 50% yield) as a white crystalline solid, m.p. 210–212 °C (decomps.); FT—IR (KBr), ν, cm^-1^: 2981.54, 2954.71, 1689.08, 1646.35; ^1^H NMR(CDCl_3_, 300 MHz) δ 3.434 (s, 3H); 3.355 (s, 6H), 3.283 (s, 3H), 1.402 (s,6*H*); ^13^C NMR (CDCl_3_, 75 MHz) δ 164.317, 160.207, 158.898, 151.033, 150.145, 93.220, 91.139, 53.872, 29.625, 29.081, 27.852, 23.318.

### Refinement

The C-bound H atoms were geometrically positioned (C–H 0.98 Å) and refined as riding, with *U*_iso_(H) = 1.2-1.5 *U*_eq_(C). In the absence of significant anomalous scatterers, 1855 Friedel pairs were merged before the final refinement.

### Crystal data

**Table:**

C_15_H_18_N_4_O_6_	*D*_x_ = 1.486 Mg m^−^^3^
*M**_r_* = 350.33	Mo *K*α radiation, λ = 0.71073 Å
Orthorhombic, *P*2_1_2_1_2_1_	Cell parameters from 985 reflections
*a* = 8.0122 (9) Å	θ = 3–25°
*b* = 11.9181 (14) Å	µ = 0.12 mm^−^^1^
*c* = 16.4037 (19) Å	*T* = 120 K
*V* = 1566.4 (3) Å^3^	Prism, white
*Z* = 4	0.21 × 0.14 × 0.12 mm
*F*(000) = 736	

### Data collection

**Table:**

Bruker SMART 1000 CCD area-detector diffractometer	1964 independent reflections
Radiation source: fine-focus sealed tube	1589 reflections with *I* > 2σ(*I*)
graphite	*R*_int_ = 0.041
φ and ω scans	θ_max_ = 27.0°, θ_min_ = 2.1°
Absorption correction: multi-scan (*SADABS*; Sheldrick, 1998)	*h* = −10→10
*T*_min_ = 0.980, *T*_max_ = 0.989	*k* = −15→15
15042 measured reflections	*l* = −20→20

### Refinement

**Table:**

Refinement on *F*^2^	Primary atom site location: structure-invariant direct methods
Least-squares matrix: full	Secondary atom site location: difference Fourier map
*R*[*F*^2^ > 2σ(*F*^2^)] = 0.047	Hydrogen site location: inferred from neighbouring sites
*wR*(*F*^2^) = 0.089	H-atom parameters constrained
*S* = 1.01	*w* = 1/[σ^2^(*F*_o_^2^) + (0.01*P*)^2^ + 2*P*] where *P* = (*F*_o_^2^ + 2*F*_c_^2^)/3
1964 reflections	(Δ/σ)_max_ < 0.001
232 parameters	Δρ_max_ = 0.20 e Å^−^^3^
0 restraints	Δρ_min_ = −0.22 e Å^−^^3^

### Special details

**Table:**

Geometry. All e.s.d.'s (except the e.s.d. in the dihedral angle between two l.s. planes) are estimated using the full covariance matrix. The cell e.s.d.'s are taken into account individually in the estimation of e.s.d.'s in distances, angles and torsion angles; correlations between e.s.d.'s in cell parameters are only used when they are defined by crystal symmetry. An approximate (isotropic) treatment of cell e.s.d.'s is used for estimating e.s.d.'s involving l.s. planes.
Refinement. Refinement of *F*^2^ against ALL reflections. The weighted *R*-factor *wR* and goodness of fit *S* are based on *F*^2^, conventional *R*-factors *R* are based on *F*, with *F* set to zero for negative *F*^2^. The threshold expression of *F*^2^ > σ(*F*^2^) is used only for calculating *R*-factors(gt) *etc*. and is not relevant to the choice of reflections for refinement. *R*-factors based on *F*^2^ are statistically about twice as large as those based on *F*, and *R*- factors based on ALL data will be even larger.

### Fractional atomic coordinates and isotropic or equivalent isotropic displacement parameters (Å^2^)

**Table:**

	*x*	*y*	*z*	*U*_iso_*/*U*_eq_	
O1	0.5464 (3)	0.52840 (19)	0.75372 (15)	0.0307 (6)	
O2	0.8328 (3)	0.8456 (2)	0.69785 (15)	0.0358 (6)	
O3	0.6235 (3)	0.7991 (2)	0.94992 (14)	0.0352 (6)	
O4	0.4987 (3)	0.59548 (19)	0.90811 (15)	0.0262 (5)	
O5	−0.0582 (3)	0.6474 (2)	0.83863 (15)	0.0333 (6)	
O6	0.0962 (3)	0.3545 (2)	1.00229 (15)	0.0306 (6)	
N1	0.6700 (4)	0.6929 (2)	0.71936 (16)	0.0247 (6)	
N2	0.6981 (4)	0.8365 (2)	0.81937 (16)	0.0251 (6)	
N4	0.0212 (3)	0.5008 (2)	0.92003 (17)	0.0242 (6)	
N5	0.3017 (3)	0.4687 (2)	0.95490 (17)	0.0242 (6)	
C1	0.5068 (4)	0.6811 (3)	0.84600 (19)	0.0229 (7)	
C2	0.5782 (4)	0.6254 (3)	0.7708 (2)	0.0250 (7)	
C3	0.7394 (4)	0.7956 (3)	0.7427 (2)	0.0254 (7)	
C4	0.6170 (4)	0.7750 (3)	0.8785 (2)	0.0252 (7)	
C5	0.3186 (4)	0.7229 (3)	0.8311 (2)	0.0276 (8)	
C6	0.2301 (4)	0.6208 (3)	0.8654 (2)	0.0242 (7)	
C7	0.3382 (4)	0.5599 (3)	0.9086 (2)	0.0236 (7)	
C8	0.1355 (4)	0.4355 (3)	0.9614 (2)	0.0237 (7)	
C9	0.0562 (4)	0.5958 (3)	0.8712 (2)	0.0258 (8)	
C10	0.7301 (5)	0.6431 (3)	0.6434 (2)	0.0334 (8)	
H10A	0.6364	0.6084	0.6143	0.050*	
H10B	0.8142	0.5858	0.6557	0.050*	
H10C	0.7799	0.7016	0.6092	0.050*	
C11	0.7816 (5)	0.9384 (3)	0.8465 (2)	0.0321 (8)	
H11A	0.7027	0.9851	0.8771	0.048*	
H11B	0.8223	0.9802	0.7990	0.048*	
H11C	0.8760	0.9186	0.8817	0.048*	
C12	0.2810 (5)	0.7474 (3)	0.7410 (2)	0.0337 (9)	
H12A	0.1620	0.7645	0.7346	0.051*	
H12B	0.3095	0.6817	0.7079	0.051*	
H12C	0.3474	0.8119	0.7229	0.051*	
C13	0.2770 (5)	0.8270 (3)	0.8822 (2)	0.0358 (9)	
H13A	0.1572	0.8428	0.8784	0.054*	
H13B	0.3400	0.8915	0.8615	0.054*	
H13C	0.3072	0.8134	0.9392	0.054*	
C14	−0.1530 (4)	0.4664 (3)	0.9244 (2)	0.0326 (8)	
H14A	−0.1596	0.3889	0.9442	0.049*	
H14B	−0.2034	0.4712	0.8701	0.049*	
H14C	−0.2133	0.5159	0.9619	0.049*	
C15	0.4309 (4)	0.3990 (3)	0.9914 (2)	0.0303 (8)	
H15A	0.5206	0.4469	1.0123	0.045*	
H15B	0.4760	0.3479	0.9501	0.045*	
H15C	0.3830	0.3553	1.0363	0.045*	

### Atomic displacement parameters (Å^2^)

**Table:**

	*U*^11^	*U*^22^	*U*^33^	*U*^12^	*U*^13^	*U*^23^
O1	0.0313 (14)	0.0252 (12)	0.0355 (13)	−0.0023 (11)	0.0011 (12)	−0.0009 (11)
O2	0.0412 (15)	0.0304 (13)	0.0357 (14)	−0.0072 (13)	0.0071 (12)	0.0047 (11)
O3	0.0416 (16)	0.0377 (14)	0.0262 (12)	−0.0048 (13)	0.0006 (12)	−0.0017 (11)
O4	0.0207 (12)	0.0275 (12)	0.0304 (12)	−0.0015 (10)	−0.0002 (10)	0.0055 (11)
O5	0.0240 (12)	0.0359 (14)	0.0399 (14)	0.0027 (12)	−0.0043 (12)	0.0057 (12)
O6	0.0257 (13)	0.0307 (13)	0.0353 (13)	−0.0014 (11)	0.0014 (12)	0.0063 (12)
N1	0.0264 (15)	0.0257 (14)	0.0221 (13)	0.0007 (13)	0.0016 (12)	0.0010 (11)
N2	0.0262 (15)	0.0225 (14)	0.0267 (14)	−0.0013 (12)	0.0014 (13)	0.0002 (12)
N4	0.0215 (14)	0.0243 (14)	0.0269 (14)	0.0011 (12)	0.0009 (12)	−0.0002 (12)
N5	0.0228 (15)	0.0228 (14)	0.0268 (14)	0.0009 (12)	0.0004 (13)	0.0025 (12)
C1	0.0207 (15)	0.0247 (16)	0.0234 (16)	−0.0003 (14)	0.0014 (14)	0.0014 (14)
C2	0.0215 (16)	0.0264 (17)	0.0272 (17)	0.0033 (15)	−0.0023 (15)	0.0004 (15)
C3	0.0258 (18)	0.0242 (16)	0.0262 (16)	−0.0007 (15)	−0.0021 (15)	0.0017 (14)
C4	0.0227 (17)	0.0257 (17)	0.0274 (17)	0.0034 (14)	0.0007 (14)	0.0008 (14)
C5	0.0196 (16)	0.0285 (18)	0.0346 (18)	0.0030 (15)	0.0035 (15)	0.0044 (15)
C6	0.0218 (16)	0.0252 (17)	0.0255 (16)	0.0005 (14)	0.0028 (14)	0.0021 (14)
C7	0.0224 (17)	0.0210 (16)	0.0275 (17)	−0.0015 (15)	0.0016 (15)	−0.0016 (14)
C8	0.0225 (17)	0.0243 (17)	0.0244 (16)	0.0015 (15)	0.0015 (14)	−0.0005 (15)
C9	0.0267 (18)	0.0261 (18)	0.0248 (17)	0.0020 (15)	0.0005 (15)	−0.0016 (15)
C10	0.039 (2)	0.0317 (19)	0.0296 (18)	0.0008 (18)	0.0025 (17)	−0.0012 (16)
C11	0.0331 (19)	0.0269 (18)	0.0363 (19)	−0.0054 (16)	0.0001 (17)	−0.0015 (16)
C12	0.0284 (19)	0.0342 (19)	0.038 (2)	0.0031 (17)	−0.0037 (17)	0.0085 (17)
C13	0.0293 (19)	0.0268 (18)	0.051 (2)	0.0024 (16)	0.0097 (19)	0.0012 (18)
C14	0.0195 (17)	0.038 (2)	0.040 (2)	−0.0035 (17)	0.0002 (16)	0.0034 (17)
C15	0.0242 (17)	0.0302 (19)	0.0364 (19)	0.0043 (16)	−0.0023 (17)	0.0091 (16)

### Geometric parameters (Å, °)

**Table:**

O1—C2	1.216 (4)	C5—C13	1.534 (5)
O2—C3	1.206 (4)	C5—C12	1.537 (5)
O3—C4	1.208 (4)	C6—C7	1.334 (5)
O4—C7	1.354 (4)	C6—C9	1.428 (5)
O4—C1	1.443 (4)	C10—H10A	0.9800
O5—C9	1.226 (4)	C10—H10B	0.9800
O6—C8	1.217 (4)	C10—H10C	0.9800
N1—C2	1.378 (4)	C11—H11A	0.9800
N1—C3	1.399 (4)	C11—H11B	0.9800
N1—C10	1.462 (4)	C11—H11C	0.9800
N2—C4	1.378 (4)	C12—H12A	0.9800
N2—C3	1.389 (4)	C12—H12B	0.9800
N2—C11	1.457 (4)	C12—H12C	0.9800
N4—C8	1.380 (4)	C13—H13A	0.9800
N4—C9	1.416 (4)	C13—H13B	0.9800
N4—C14	1.457 (4)	C13—H13C	0.9800
N5—C7	1.358 (4)	C14—H14A	0.9800
N5—C8	1.393 (4)	C14—H14B	0.9800
N5—C15	1.456 (4)	C14—H14C	0.9800
C1—C2	1.513 (4)	C15—H15A	0.9800
C1—C4	1.522 (5)	C15—H15B	0.9800
C1—C5	1.607 (5)	C15—H15C	0.9800
C5—C6	1.516 (5)		
			
C8···O2^i^	2.835 (4)	C3···O5^ii^	2.868 (4)
			
C7—O4—C1	105.6 (3)	O6—C8—N5	121.0 (3)
C2—N1—C3	123.8 (3)	N4—C8—N5	115.8 (3)
C2—N1—C10	117.4 (3)	O5—C9—N4	120.0 (3)
C3—N1—C10	117.2 (3)	O5—C9—C6	126.6 (3)
C4—N2—C3	124.3 (3)	N4—C9—C6	113.4 (3)
C4—N2—C11	116.4 (3)	N1—C10—H10A	109.5
C3—N2—C11	117.3 (3)	N1—C10—H10B	109.5
C8—N4—C9	126.8 (3)	H10A—C10—H10B	109.5
C8—N4—C14	116.9 (3)	N1—C10—H10C	109.5
C9—N4—C14	116.3 (3)	H10A—C10—H10C	109.5
C7—N5—C8	118.5 (3)	H10B—C10—H10C	109.5
C7—N5—C15	122.2 (3)	N2—C11—H11A	109.5
C8—N5—C15	119.1 (3)	N2—C11—H11B	109.5
O4—C1—C2	106.4 (3)	H11A—C11—H11B	109.5
O4—C1—C4	107.4 (3)	N2—C11—H11C	109.5
C2—C1—C4	112.9 (3)	H11A—C11—H11C	109.5
O4—C1—C5	106.5 (3)	H11B—C11—H11C	109.5
C2—C1—C5	111.5 (3)	C5—C12—H12A	109.5
C4—C1—C5	111.7 (3)	C5—C12—H12B	109.5
O1—C2—N1	121.7 (3)	H12A—C12—H12B	109.5
O1—C2—C1	121.7 (3)	C5—C12—H12C	109.5
N1—C2—C1	116.4 (3)	H12A—C12—H12C	109.5
O2—C3—N2	121.8 (3)	H12B—C12—H12C	109.5
O2—C3—N1	120.8 (3)	C5—C13—H13A	109.5
N2—C3—N1	117.3 (3)	C5—C13—H13B	109.5
O3—C4—N2	122.4 (3)	H13A—C13—H13B	109.5
O3—C4—C1	122.6 (3)	C5—C13—H13C	109.5
N2—C4—C1	114.7 (3)	H13A—C13—H13C	109.5
C6—C5—C13	110.2 (3)	H13B—C13—H13C	109.5
C6—C5—C12	114.7 (3)	N4—C14—H14A	109.5
C13—C5—C12	109.2 (3)	N4—C14—H14B	109.5
C6—C5—C1	97.7 (3)	H14A—C14—H14B	109.5
C13—C5—C1	111.8 (3)	N4—C14—H14C	109.5
C12—C5—C1	112.9 (3)	H14A—C14—H14C	109.5
C7—C6—C9	119.0 (3)	H14B—C14—H14C	109.5
C7—C6—C5	109.3 (3)	N5—C15—H15A	109.5
C9—C6—C5	130.4 (3)	N5—C15—H15B	109.5
C6—C7—O4	116.3 (3)	H15A—C15—H15B	109.5
C6—C7—N5	126.4 (3)	N5—C15—H15C	109.5
O4—C7—N5	117.3 (3)	H15A—C15—H15C	109.5
O6—C8—N4	123.1 (3)	H15B—C15—H15C	109.5
			
C7—O4—C1—C2	−99.4 (3)	C4—C1—C5—C13	−22.4 (4)
C7—O4—C1—C4	139.5 (3)	O4—C1—C5—C12	−141.8 (3)
C7—O4—C1—C5	19.7 (3)	C2—C1—C5—C12	−26.1 (4)
C3—N1—C2—O1	−166.1 (3)	C4—C1—C5—C12	101.2 (3)
C10—N1—C2—O1	−0.8 (5)	C13—C5—C6—C7	−101.6 (3)
C3—N1—C2—C1	19.0 (5)	C12—C5—C6—C7	134.8 (3)
C10—N1—C2—C1	−175.7 (3)	C1—C5—C6—C7	15.1 (4)
O4—C1—C2—O1	35.2 (4)	C13—C5—C6—C9	64.9 (5)
C4—C1—C2—O1	152.8 (3)	C12—C5—C6—C9	−58.7 (5)
C5—C1—C2—O1	−80.6 (4)	C1—C5—C6—C9	−178.4 (4)
O4—C1—C2—N1	−149.9 (3)	C9—C6—C7—O4	−172.6 (3)
C4—C1—C2—N1	−32.3 (4)	C5—C6—C7—O4	−4.3 (4)
C5—C1—C2—N1	94.4 (3)	C9—C6—C7—N5	4.8 (5)
C4—N2—C3—O2	−168.0 (3)	C5—C6—C7—N5	173.1 (3)
C11—N2—C3—O2	−4.5 (5)	C1—O4—C7—C6	−10.5 (4)
C4—N2—C3—N1	11.2 (5)	C1—O4—C7—N5	171.8 (3)
C11—N2—C3—N1	174.8 (3)	C8—N5—C7—C6	−2.2 (5)
C2—N1—C3—O2	172.2 (3)	C15—N5—C7—C6	171.9 (3)
C10—N1—C3—O2	6.9 (5)	C8—N5—C7—O4	175.2 (3)
C2—N1—C3—N2	−7.0 (5)	C15—N5—C7—O4	−10.7 (5)
C10—N1—C3—N2	−172.3 (3)	C9—N4—C8—O6	−179.9 (3)
C3—N2—C4—O3	158.8 (3)	C14—N4—C8—O6	−2.3 (5)
C11—N2—C4—O3	−4.9 (5)	C9—N4—C8—N5	1.2 (5)
C3—N2—C4—C1	−26.5 (5)	C14—N4—C8—N5	178.7 (3)
C11—N2—C4—C1	169.9 (3)	C7—N5—C8—O6	−179.8 (3)
O4—C1—C4—O3	−32.7 (4)	C15—N5—C8—O6	5.9 (5)
C2—C1—C4—O3	−149.7 (3)	C7—N5—C8—N4	−0.9 (5)
C5—C1—C4—O3	83.7 (4)	C15—N5—C8—N4	−175.1 (3)
O4—C1—C4—N2	152.5 (3)	C8—N4—C9—O5	−179.0 (3)
C2—C1—C4—N2	35.5 (4)	C14—N4—C9—O5	3.5 (5)
C5—C1—C4—N2	−91.1 (3)	C8—N4—C9—C6	1.3 (5)
O4—C1—C5—C6	−20.8 (3)	C14—N4—C9—C6	−176.3 (3)
C2—C1—C5—C6	94.9 (3)	C7—C6—C9—O5	176.2 (3)
C4—C1—C5—C6	−137.8 (3)	C5—C6—C9—O5	10.8 (6)
O4—C1—C5—C13	94.6 (3)	C7—C6—C9—N4	−4.1 (5)
C2—C1—C5—C13	−149.7 (3)	C5—C6—C9—N4	−169.5 (3)

Symmetry codes: (i) −*x*+1, *y*−1/2, −*z*+3/2; (ii) *x*+1, *y*, *z*.
